# Vasculitic fasciitis characterizes a distinct subset of vasculitic myopathy with interferon-gamma signature

**DOI:** 10.1007/s00401-025-02969-1

**Published:** 2025-12-24

**Authors:** Nikolas Ruffer, Iago Pinal-Fernandez, Corinna Preusse, Andrew L. Mammen, Marie-Therese Holzer, Felix Kleefeld, Hans-Hilmar Goebel, Maria Casal-Dominguez, Katherine Pak, Ina Kötter, Jeffrey Siefert, Christian Furth, Felix Feldhaus, Norman Görl, Franziska Fieber, Rieke Alten, Tobias B. Huber, Vincent Casteleyn, Andreas Roos, Martin Krusche, Udo Schneider, José César Milisenda, Werner Stenzel

**Affiliations:** 1 III. Department of Medicine, University Medical Center Hamburg-Eppendorf, Hamburg, Germany; 2 Department of Neuropathology, Charité-Universitätsmedizin Berlin, corporate member of Freie Universität Berlin and Humboldt-Universität zu Berlin, Charitéweg 1-Virchowweg 15, 10117 Berlin, Germany; 3 Muscle Disease Section, National Institute of Arthritis and Musculoskeletal and Skin Diseases, National Institutes of Health, Bethesda, MD USA; 4 Department of Neurology, School of Medicine, Johns Hopkins University, Baltimore, MD USA; 5 Clinic for Neurology With Experimental Neurology, Charité-Universitätsmedizin Berlin, corporate member of Freie Universität Berlin and Humboldt-Universität zu Berlin, Berlin, Germany; 6 Clinic for Pediatrics With Neurology, Charité-Universitätsmedizin Berlin, corporate member of Freie Universität Berlin and Humboldt-Universität zu Berlin, Berlin, Germany; 7 Department of Medicine, School of Medicine, Johns Hopkins University, Baltimore, MD USA; 8 Department of Neurology, Heimer Institute for Muscle Research, University Hospital Bergmannsheil, Ruhr University Bochum, Bochum, Germany; 9 Department of Neurology, BG University Hospital Bergmannsheil, Ruhr University Bochum, Bochum, Germany; 10 Department of Nuclear Medicine, Charité-Universitätsmedizin Berlin, corporate member of Freie Universität Berlin and Humboldt-Universität zu Berlin, Berlin, Germany; 11 Department of Radiology, Charité-Universitätsmedizin Berlin, corporate member of Freie Universität Berlin and Humboldt-Universität zu Berlin, Berlin, Germany; 12 Rheumazentrum Nordwest, Wismar, Germany; 13 Department of Internal Medicine, Rheumatology and Immunology, Klinikum Südstadt Rostock, Rostock, Germany; 14 Department of Internal Medicine and Rheumatology, Schlosspark-Klinik, Berlin, Germany; 15 Department of Rheumatology and Clinical Immunology, Charité-Universitätsmedizin Berlin, corporate member of Freie Universität Berlin and Humboldt-Universität zu Berlin, Berlin, Germany; 16 Department of Neuropediatrics, Developmental Neurology and Social Pediatrics, Centre for Neuromuscular Disorders in Children, University Children’s Hospital Essen, University of Duisburg-Essen, Essen, Germany; 17 Department of Rheumatology, Clinical Immunology and Osteology, Immanuel Hospital Berlin, Berlin, Germany; 18 Muscular and Inherited Metabolic Disorders Research Laboratory, Internal Medicine Department, Hospital Clínic de Barcelona, Barcelona, Spain

**Keywords:** Polyarteritis nodosa, Vasculitis, Fasciitis, Myositis, Myopathology

## Abstract

Vasculitic myopathy (VM) represents a nonspecific manifestation of various vasculitic syndromes that presents with myalgia, leg tenderness, and muscle weakness. Most cases of VM develop in the context of polyarteritis nodosa (PAN), antineutrophil cytoplasmic antibody (ANCA)-associated vasculitis, or rheumatoid vasculitis, or manifest as single organ vasculitis. Muscle inflammation in VM is poorly understood, and most studies essentially report the presence of vasculitic changes demonstrated by microscopic analysis of skeletal muscle tissue. However, no detailed characterization or in-depth analyses have been performed so far. We studied the clinicopathologic phenotype of 12 patients and analyzed the gene expression profiles of 36 patients with VM in the context of negative ANCA test results. ANCA-negative VM typically presents with myalgia involving the lower extremities, accompanied by cutaneous manifestations, constitutional symptoms, and marked systemic inflammation. Half of the patients (6/12; 50.0%) met the classification criteria for ‘classic PAN’. Serum creatine kinase (CK) activity was normal in most cases (10/12; 83.3%). The presence of skeletal muscle vasculitis was confirmed by histopathology in 9/12 (75.0%) cases. Specifically, ANCA-negative VM predominantly affected small arteries and was consistently associated with small vessel involvement of the epimysial fascia (9/10; 90.0%), which was termed ‘vasculitic fasciitis’ (VF). Signs of necrotizing vasculitis were occasionally noted (3/9; 33.3%). Mild endomysial fibrosis and muscle fiber necrosis could be compatible with a rather acute disease onset and may account for normal or slightly elevated levels of serum CK activity, respectively. Additionally, nonspecific myopathic changes such as capillary vessel mural thickening and variations of muscle fiber size were detected in all analyzed muscle biopsy specimens. Immunohistochemical studies revealed perifocal sarcolemmal upregulation of major histocompatibility complex class I in the majority of cases (10/11; 90.9%). RNA sequencing of muscle biopsies from 36 ANCA-negative VM patients, compared to 37 healthy controls and 649 samples from other inflammatory myopathies, revealed that ANCA-negative VM is characterized by a dominant interferon-gamma-driven immune response, broad cytokine activation including tumor necrosis factor-related genes, and selective upregulation of angiogenesis- and endothelium-associated transcripts. Our histomorphologic analysis highlights a distinct histopathological pattern of VF that is characteristic for muscle involvement in ANCA-negative vasculitis. Furthermore, we provide evidence for a specific molecular signature that gives new insights into the pathogenesis and treatment of ANCA-negative VM.

**Supplementary Information:**

The online version contains supplementary material available at 10.1007/s00401-025-02969-1.

## Introduction

Primary vasculitides are systemic immune-mediated disorders marked by inflammation of multiple organ systems including the skeletal muscle—the latter is referred to as ‘vasculitic myopathy’ (VM). VM typically presents with myalgia, leg tenderness, and muscle weakness [9, 24, 28, 47, 54, 60, 62]. In addition, constitutional symptoms such as fever, cutaneous manifestations, or sensory–motor symptoms including painful mononeuritis secondary to concomitant neuropathy can also develop [9, 17, 24, 28, 47, 54, 56, 60].

VM represents a nonspecific manifestation of various vasculitic syndromes, and diagnostic considerations are largely influenced by clinical context (e.g., autoantibody profile, extramuscular organ involvement, histomorphology). Most cases of VM develop in the setting of polyarteritis nodosa (PAN), myeloperoxidase-specific antineutrophil cytoplasmic antibody (MPO-ANCA)-associated vasculitis (AAV), rheumatoid vasculitis, or manifest as single organ vasculitis that predominantly affects medium- or small-sized arteries, respectively [9, 27, 39, 47, 62].

Laboratory tests usually demonstrate elevated levels of inflammatory markers, while serum creatine kinase (CK) activity is normal in most cases of VM [9, 14, 15, 27, 30, 39, 47, 62]. The magnetic resonance imaging (MRI) morphology of VM includes signs of fasciitis and diffuse or patchy intramuscular hyperintensities on T2- or short-tau inversion recovery (STIR) sequences that indicate muscle edema (secondary to vasculitis) [9, 62]. The latter finding can resemble signal alterations associated with idiopathic inflammatory myopathies (IIM). In this context, the term ‘MRI myositis sine myositis’ [3] has been coined to capture the stereotypical nature of VM. For contrast-enhanced MRI, a ‘cotton-wool appearance’ in the vicinity of branching intramuscular blood vessels has been reported [20, 23]. Furthermore, [^18^F] fluorodeoxyglucose positron emission tomography/computed tomography ([^18^F]-FDG PET/CT) imaging studies recently described a specific pattern of increased radiotracer uptake in the skeletal muscle tissue and intramuscular arteries (so-called ‘dirty muscle sign’) that is associated with active VM in PAN [13, 14, 42, 55]. However, the confirmation of destructive inflammation of the vessel wall demonstrated by histopathologic evaluation of a suitable muscle biopsy specimen still remains the gold standard for the diagnosis of VM.

The pathogenesis of muscle inflammation in VM is poorly understood. Most studies essentially report the presence of vasculitic changes demonstrated by muscle biopsy, but fail to capture the histomorphologic phenotype of (ANCA-negative) VM. Features of perivascular inflammation or fibrinoid vessel wall necrosis have been reported as the most common morphologic patterns in heterogeneous cohorts of VM [47, 62]. Also, fasciitis has been occasionally described in cases of PAN [1, 17, 19, 36, 40, 51, 61]. One retrospective study of 13 muscle biopsy specimens that also included immunohistochemical analyses reported a pattern of patchy or diffuse upregulation of major histocompatibility complex (MHC) class I involving the sarcolemma (and sarcoplasm) [47]. In addition, complement deposits on endomysial capillaries, necrotic and regenerating myofibers, as well as CD4^+^ inflammatory infiltrates, were noted in the majority of cases [47]. However, the noted upregulation of MHC class I, complement deposits, and myofiber damage in VM have been reported to be less pronounced compared to IIM according to another study [38]. The rather mild degree of muscle fiber degeneration may account for normal levels of serum CK activity, which is typically found in VM [62].

Of note, both studies [38, 47] analyzed muscle biopsy specimens of a heterogeneous cohort that included cases of AAV and a few samples from ANCA-negative cases. Interestingly, a recent systematic review [62] suggested that VM in PAN (not associated with ANCA) and AAV differs in clinical presentation, with myalgia, muscle weakness, and distal lower extremity involvement being more pronounced in PAN. Whether these differences in clinical phenotype are reflected by histomorphology, as recently demonstrated in vasculitic neuropathy (VN) [63], is unclear.

Historically, VM has received limited attention by neuropathologists and clinicians in comparison to VN, despite the well-known clinical value of confirming the diagnosis of systemic vasculitis through muscle biopsy [32, 38, 39, 47, 50, 54, 65, 70, 72]. This issue is of particular interest, since both conditions are closely related as demonstrated by multiple studies [5, 27, 39, 49, 58, 66, 71].

The aim of our study was to investigate the phenotype of VM in a homogeneous cohort of ANCA-negative vasculitis based on skeletal muscle biopsy specimens and the retrospective analysis of patient charts. Multidimensional morphologic and immune profiling was carried out to clarify the pathophysiology of muscle inflammation in this underrecognized manifestation of vasculitis.

## Methods

### Patients and muscle biopsy specimens

The pathology reports of skeletal muscle biopsies from the Department of Neuropathology at Charité-Universitätsmedizin Berlin (Berlin, Germany) between 2008 and 2023 were retrospectively reviewed. Muscle biopsy specimens that were obtained for suspected inflammatory muscle disease were selected for subsequent histopathologic evaluation and patient chart review.

The inclusion of muscle biopsy specimens was based on the following criteria (Table 1): (A1) biopsy-proven skeletal muscle vasculitis and/or (A2) imaging findings (MRI and whole body [^18^F]-FDG PET imaging) indicating active vasculitis and concomitant signs of muscle inflammation as reported in the literature [13, 14, 16, 23, 26, 42, 55], (B) negative ANCA test results (proteinase 3-specific ANCA, MPO-ANCA), (C) laboratory findings indicating systemic inflammation (elevated concentration of C-reactive protein [CRP]), and (D) clinical diagnosis of VM in the context of systemic vasculitis based on patient chart review. Clinical data at the time of the muscle biopsy procedure were retrieved and evaluated by two rheumatologists (NR, US).

**Table 1:** Inclusion criteria applied in this study for the classification of cases as ‘ANCA-negative vasculitic myopathy’

(A) Muscle inflammation in the context of systemic vasculitis:
(A1) Confirmation of skeletal muscle vasculitis by muscle biopsy and/or
(A2) Imaging findings: [^18^F]-FDG PET imaging indicating active vasculitis and MRI consistent with active muscle inflammation
(B) Autoantibody testing: negative test results for MPO-ANCA and PR3-ANCA
(C) Laboratory: systemic inflammation indicated by elevated concentration of C-reactive protein
(D) Clinical diagnosis of vasculitic myopathy based on patient chart review (e.g., exclusion of other causes)

Inclusion and classification of cases as ‘ANCA-negative vasculitic myopathy’ was based on the presence of all criteria (A–D)

*[*^*18*^*F]-FDG PET* [^18^F] fluorodeoxyglucose positron emission tomography, *ANCA* antineutrophil cytoplasmic antibody, *MPO* myeloperoxidase, *MRI* magnetic resonance imaging, *PR3* proteinase 3

Additionally, one case of ANCA-negative VM [57] from the University Medical Center Hamburg–Eppendorf was included in this study.

For immune profiling, muscle biopsy specimens were analyzed via quantitative real-time polymerase chain reaction (qRT-PCR) and bulk RNA sequencing (RNAseq).

Muscle biopsy specimens from non-disease controls (NDC; *n* = 6) were analyzed via qRT-PCR for comparative analysis. NDC patients presented with rather nonspecific symptoms such as subjective muscle weakness and pain, which could not be clearly defined in clinical terms. Muscle biopsies were obtained from these patients in the context of diagnostic evaluations. Routine histopathological and diagnostic analyses did not reveal any abnormalities of skeletal muscle tissue, including the absence of inflammatory changes. Moreover, there were no associated laboratory or clinical findings indicative of muscle disease, such as elevated serum creatine kinase (CK) activity or the presence of autoantibodies.

RNAseq of 10 ANCA-negative VM muscle biopsy specimens from Charité-Universitätsmedizin Berlin (‘Berlin cohort’) was performed at the National Institutes of Health (NIH) (Bethesda, Maryland, USA). Additionally, 26 ANCA-negative VM muscle biopsy specimens from the Muscular and Inherited Metabolic Disorders Research Laboratory at the Hospital Clínic de Barcelona (Barcelona, Spain) (‘Barcelona cohort’) that met the above-defined inclusion criteria (Table 1) were also analyzed with RNAseq at the NIH. Muscle biopsy specimens from patients with different types of IIM and genetic myopathies were used for comparative analyses as previously described [2, 43–46]. Of the 37 biopsies from healthy comparators, 16 were from histologically normal samples obtained at Johns Hopkins and at the Hospital Clínic de Barcelona for clinical purposes, and the remaining 21 samples were obtained from healthy volunteers.

The Charité-Universitätsmedizin Berlin ethics committee had granted ethical approval (EA2/163/17 and EA1/019/22) for this study.

### Tissue preparation

11/12 ANCA-negative VM muscle biopsy specimens were cryopreserved immediately after removal at − 80 °C. Cryostat sections (8 µm thick) were stained according to local standardized procedures [48] and international recommendations [11, 68]. One (1/12) of the ANCA-negative VM muscle biopsy specimens was fixed and embedded in paraffin, which limited staining procedures and, therefore, evaluation of the tissue including gene expression analysis.

Diagnostic conventional histology and enzyme histochemistry reactions (hematoxylin and eosin [H&E], Gömöri trichrome [Gö], Elastic van Gieson [EvG], acid phosphatase [AcP], nonspecific esterase [NSE], cytochrome c oxidase/succinate dehydrogenase [COX/SDH], SDH, ATPases pH [4.3, 4.6, and 9.4]) were performed. Immunohistochemical staining procedures were carried out according to standardized protocols. The following primary antibodies were used: MHC class I, MHC class II, C5b-9, CD4, CD8, CD20, CD31, CD45, CD68, CD138, multiple myeloma oncogene 1 (MUM1), intercellular adhesion molecule (ICAM), vascular endothelial growth factor (VEGF), platelet-derived growth factor receptor beta (PDGFRB). Supplementary Table S1 details the primary antibodies, dilutions, species, companies, and purchase numbers used.

Appropriate positive and negative controls were used where necessary, and a physiological control or normal muscle tissues were used as a negative control for all reactions, as outlined previously [48].

### Morphologic analysis

Based on conventional and immunohistochemical stains, we performed a qualitative analysis of the following findings: location of inflammatory infiltrates, skeletal muscle vasculitis (present/absent), features of necrotizing vasculitis (present/absent), involved vessel caliber (small vessel vasculitis [SVV], medium vessel vasculitis [MVV]), perivascular infiltrates (present/absent), fasciitis (present/absent), occlusive vasculopathy (present/absent), muscle fiber necrosis (present/absent), variations of muscle fiber size (present/absent), signs of neurogenic damage (present/absent), sarcolemmal or capillary complement depositions (present/absent), capillary vessel mural thickening (present/absent), endomysial fibrosis (present/absent), sarcolemmal staining pattern of MHC class I and MHC class II (upregulation on myofibres present/absent), and pericapillary staining of PDGFRB for the identification of pericytes (present/absent).

Skeletal muscle vasculitis was defined as (a) vessel wall infiltration by inflammatory cells and (b) at least one additional finding of the following (adapted from [63]): evidence of vessel wall destruction based on (b1) vessel wall disruption, (b2) fibrinoid necrosis, (b3) vessel thrombosis, (b4) vessel scarring/fibrosis, or (b5) vessel wall separation, fragmentation, and damage by inflammatory cells.

Semiquantitative analysis of the following features was performed (+ mildly positive/present; ++ moderately positive/present; +++ strongly positive/present; − negative/not present): CD68 and CD31.

Quantitative assessment of inflammatory infiltrates with counting of 10 high power fields (HPFs) based on the microscope used and the respective oculars (Olympus WH10x-H/22 ≙ 0.16 mm^2^) was performed for: CD3, CD8, and CD45.

### Transmission electron microscopy (TEM)

Ultrastructural evaluation of ANCA-negative VM using TEM was performed in two cases. Muscle biopsy specimens were fixed and embedded according to standard protocols as previously described [21]. Briefly, 70 nm ultrathin sections were cut using an ultramicrotome and an Ultra 35° diamond knife (Diatome), stretched with xylene vapor, collected onto pioloform-coated slot grids, and then stained with lead citrate. Standard TEM was performed using a Zeiss 901 microscope in conjunction with a 2k CCD camera (TRS).

### RNA extraction and quantitative real-time polymerase chain reaction

Quantitative real-time polymerase chain reaction was performed to analyze the gene expression profiles as previously reported [21, 48]. TRIzol/chloroform was used for total RNA extraction. Subsequently, reverse transcription and cDNA synthesis were carried out using the High-Capacity cDNA Archive Kit (Applied Biosystems, Foster City, California, USA) according to the manufacturer’s protocol. An Applied Biosystems™ QuantStudio™ 6 Flex Real-Time PCR System (ThermoFisher, Waltham, Massachusetts, USA; running conditions: 95 °C 0:20, 95 °C 0:01, 60 °C 0:20, 40 cycles) was used for qRT-PCR reactions. Target genes were normalized to the expression of housekeeping gene *PGK1* Hs99999906_m1. Genes analyzed were: *C1QA* Hs00706358_s1, *CD28* Hs01007422_m1, *IFNG* Hs00989291_m1, *IL10* Hs00961622_m1, *IL1B* Hs01555410_m1, *IL5* Hs01548712_g1, *IL6* Hs00985639_m1, *SIGLEC1* Hs00224991_m1, *STAT3* Hs00374280_m1, and *TNFA* Hs00174128_m1.

Gene expression is illustrated by dCT values showing expression in ANCA-negative VM patients and NDC. Some patients had no detectable gene expression and a dCT value of 20 was added for visualization purposes of the whole cohort.

### RNA sequencing

Bulk RNA sequencing was conducted on frozen muscle biopsy specimens, following previously established protocols [2, 43–46]. Muscle samples were immediately flash-frozen upon biopsy and stored at − 80 °C at each participating center. They were then transported on dry ice to the NIH, where they were uniformly processed. RNA extraction was performed using TRIzol. Libraries were prepared either using the NeoPrep system with the TruSeq Stranded mRNA Library Prep protocol (Illumina, San Diego, CA) or with the NEBNext Poly(A) mRNA Magnetic Isolation Module and Ultra™ II Directional RNA Library Prep Kit for Illumina (New England BioLabs, ref. #E7490 and #E7760).

### Statistical analysis

For qRT-PCR analysis, sample sizes were not based on an a priori power calculation, but based on previous studies [21]. Data are presented as violin plots. Gene expression was tested by the Mann–Whitney *U* test or Kruskal–Wallis test, followed by Bonferroni–Dunn correction for multiple comparisons. The level of significance was set at *p* < 0.05. GraphPad Prism 9.02 software (GraphPad Software, Inc., La Jolla, CA, USA) was used for statistical analysis and visualization.

For RNAseq analysis, sequencing reads were demultiplexed using bcl2fastq/2.20.0 and preprocessed using fastp/0.21.0. The abundance of each gene was determined using Salmon/1.5.2. Counts were normalized using the trimmed means of M values (TMM) from edgeR/3.34.1 for graphical analysis. Differential expression was performed using limma/3.48.3. Pathway enrichment analysis was performed using clusterProfiler (v4.12.6) to perform gene set enrichment analysis (GSEA) against the Hallmark gene sets from the Molecular Signatures Database (MSigDB). Results were visualized using enrichplot (v1.24.4). Gene lists were curated from the HUGO Gene Nomenclature Committee (HGNC). Relative expression was expressed as the log2 fold-change (logFC). Where applicable, *p* values were adjusted for multiple comparisons using the Benjamini–Hochberg procedure, with a false discovery rate threshold of *q* < 0.05 considered statistically significant.

## Results

### Clinical features of ANCA-negative vasculitic myopathy

The studied cohort included patients with ANCA-negative VM in the context of systemic vasculitis, identified retrospectively at the Department of Neuropathology at Charité-Universitätsmedizin Berlin (Germany). Additionally, one muscle biopsy specimen [57] from the University Medical Center Hamburg–Eppendorf was included. In total, 12 muscle biopsies were analyzed. All muscle biopsies were obtained from the lower extremity, with the *gastrocnemius* muscle being the most frequent biopsy site (8/12; 75.0%). 10/12 (83.3%) muscle biopsy specimens also included fascial tissue.

Median age at the time of muscle biopsy was 56.5 years (range: 23–71 years). 7/12 of the patients were male. Half of the patients (6/12) met the 1990 American College of Rheumatology (ACR) classification criteria for ‘classic PAN’ [34] (Table 2). In addition to myalgia (12/12; 100%), skin involvement (6/12; 50.0%) and constitutional symptoms such as fever (8/12; 75.0%) or weight loss (2/12; 16.6%) were common. Interestingly, 2/12 patients reported the presence of testicular pain, which represents a relatively specific symptom of PAN. Notably, diagnostic angiography was not performed in any of the patients.

**Table 2:** Clinical and muscle biopsy features of patients with ANCA-negative vasculitic myopathy in the Berlin cohort

Median age (range)	56.5 years (23–71)
Sex ratio (male/female)	7/5
Clinical features	
American College of Rheumatology 1990 classification criteria for polyarteritis nodosa	6/12 (50.0%)
Weight loss	2/12 (16.6%)
Livedo reticularis	3/12 (25.0%)
Testicular pain or tenderness	2/12 (16.6%)
Myalgia	12/12 (100%)
Neuropathy	3/12 (25.0%)
Diastolic blood pressure > 90 mmHg	2/12 (16.6%)
Elevated creatinine	1/12 (8.3%)
Hepatitis B virus	0/10 (0%)
Arteriographic abnormality	n/a
Biopsy of small- or medium-sized artery containing polymorphonuclear neutrophils	9/12 (75.0%)
Calf swelling	4/12 (25.0%)
Fever	8/12 (75.0%)
Skin involvement	6/12 (50.0%)
Sensory deficits	3/12 (25.0%)
Arthralgia	5/12 (41.6%)
Laboratory features	
Median C-reactive protein concentration	120.5 mg/L
Median (range) serum creatine kinase activity	41 U/L (28–588)
Elevated serum creatine kinase activity	2/12 (16.6%)
Median creatinine concentration	0.76 mg/dl
Myeloperoxidase-specific antineutrophil cytoplasmic antibody	0/12 (0%)
Proteinase 3-specific antineutrophil cytoplasmic antibody	0/12 (0%)
Hepatitis C virus	0/10 (0%)
Human immunodeficiency virus	0/9 (0%)
Imaging features	
Intramuscular hyperintensities on magnetic resonance imaging (T2- or short-tau inversion recovery sequences)	10/10 (100%)
Fascial involvement on magnetic resonance imaging	7/7 (100%)
Radiotracer uptake in peri- and/or intramuscular arteries	5/5 (100%)
Upper extremity involvement	2/5 (40.0%)
Lower extremity involvement	11/11 (100%)
Muscle biopsy features	
Biopsy site	
*Gastrocnemius* muscle	8/12 (75.0%)
*Quadriceps femoris* muscle	4/12 (25.0%)
Presence of fascial tissue	10/12 (83.3%)

Laboratory investigations demonstrated highly elevated levels of inflammatory markers (median CRP concentration 120.5 mg/L, normal range < 5 mg/L), while serum CK activity was normal in the majority of cases (10/12; 83.3%) and mildly elevated in 2/12 cases (maximum serum CK activity 588 U/L, normal range < 167 U/L). Serologic studies did not disclose a virus infection (hepatitis B virus, hepatitis C virus, human immunodeficiency virus) that would indicate secondary vasculitis in any of the patients.

MRI demonstrated diffuse or patchy hyperintensities on T2 sequences within the skeletal muscle of the lower extremity indicating muscle inflammation in all patients (10/10; 100%), when performed, and was used to guide the muscle biopsy procedure in these patients (Fig. 2). MRI data of fascial involvement indicated fasciitis in all available cases (7/7; 100%). Whole body [^18^F]-FDG PET imaging (4 PET/CT studies, 1 PET/MRI study) additionally revealed upper extremity involvement in 2/5 (40.0%) cases, in addition to the ubiquitous presence of inflammatory lesions of the lower extremity. Pathologic radiotracer uptake in peri- and/or intramuscular arteries was observed in 5/5 cases (100%) in whom whole body [^18^F]-FDG PET imaging was performed.

Table 2 displays clinical and muscle biopsy features of the muscle biopsy specimens derived from ANCA-negative patients with ANCA-negative VM.

### Morphologic features of ANCA-negative vasculitic myopathy

**Fig. 1:**
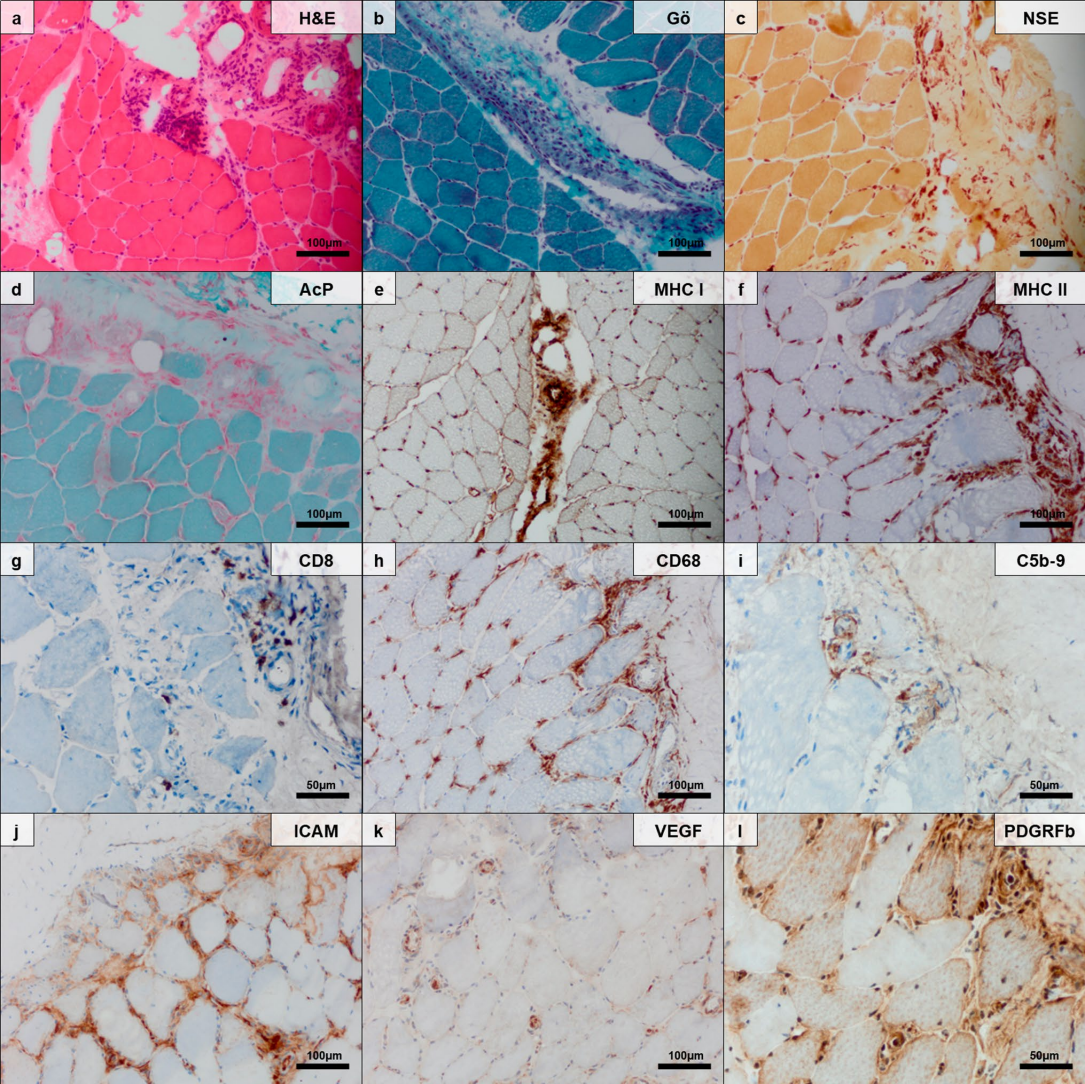
Histomorphology of a representative muscle biopsy specimen of vasculitic myopathy in ANCA-negative small vessel vasculitis. Skeletal muscle tissue and adjacent epimysial fascia with numerous arterioles showing inflammatory infiltrates of the vessel wall structures with variable density and degree of vessel destruction/invasion. Note a minimal degree of perimysial leukocytic infiltration that extends into the endomysium close to the vasculitis. Muscle fibers were hypotrophic in some instances, but features of perifascicular atrophy or a gradient of atrophy towards the centrofascicular regions were absent (hematoxylin and eosin staining) (**a**). Leukocytic infiltrates of the vessel wall and perimysial enlargement with edematous changes of a perimysial arteriole, which is partially cut in a longitudinal way. The adjacent myofibers show some degree of atrophy without an obvious gradient (Gömöri trichrome preparation) (**b**). Macrophages at the skeletal muscle–fascia interface accumulating around small vessels and partially extending into the perimysium (nonspecific esterase and acid phosphatase enzyme histochemical preparations) (**c**, **d**). Only single fibers show sarcolemmal major histocompatibility complex (MHC) class I positivity in the perifascicular region, while MHC class II does not stain the sarcolemma of myofibers. Note that macrophages in the fascia are prominently stained by both antibodies (MHC class I and MHC class II immunohistochemistry) (**e**, **f**). Single CD8^+^ cells are detectable around some of the epimysial arterioles (CD8 immunohistochemistry) (**g**). Numerous CD68^+^ macrophages at the muscle–fascia interface accumulating around small vessels (CD68 immunohistochemistry) (**h**). Membrane attack complex does not stain any capillaries or the sarcolemma within the skeletal muscle fascicles, but the epimysial arterioles are strongly stained (C5b-9 immunohistochemistry) (**i**). Staining of intercellular adhesion molecule (ICAM), vascular endothelial growth factor (VEGF), and platelet-derived growth factor receptor beta (PDGFRB) strongly highlights the activated vessel wall structures as well as activated pericytes at the muscle fascia interface (ICAM, VEGF, PDGFRB immunohistochemistry) (**j**–**l**)

Histomorphologic analysis of the muscle biopsy specimens demonstrated vasculitis in 9/12 cases (75.0%), including 8/9 cases of skeletal muscle vasculitis and 1/9 case of vasculitis confined to the fascia (Fig. 1). Predominant affection of small arteries (SVV) was observed in the majority of cases (6/9; 66.6%), and medium-sized arteries (MVV) were less frequently (3/9 cases; 33.3%) affected. A subset of muscle biopsy specimens exhibited a pattern of necrotizing vasculitis (3/9; 33.3%).

**Fig. 2:**
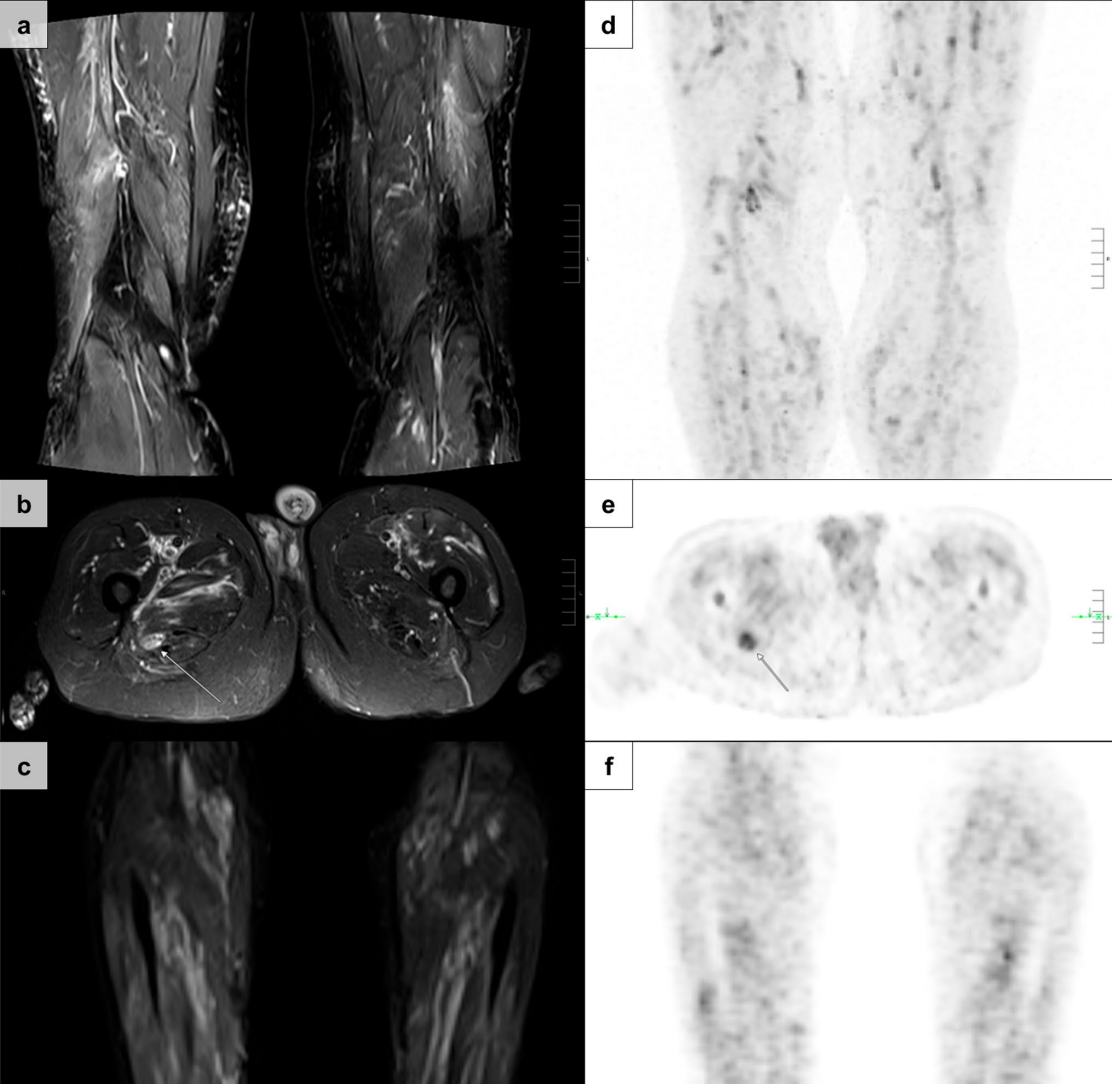
Imaging findings of vasculitic myopathy in ANCA-negative vasculitis. T2-weighted magnetic resonance imaging findings of multifocal hyperintensities pronounced in the vicinity of intramuscular arteries, consistent with edematous lesions secondary to vasculitis (**a**–**c**). Corresponding whole body [^18^F] fluorodeoxyglucose positron emission tomography imaging demonstrates increased radiotracer uptake in peri- and intramuscular arteries of the lower extremities—referred to as the ‘dirty muscle sign’ (**d**–**f**)

Muscle biopsy specimens without biopsy confirmation of vasculitis (3/12; 25.0%) harbored nonspecific myopathic features, including variations of muscle fiber size and capillary vessel mural thickening. In these cases (Fig. 2), the diagnosis of VM was based on MRI, indicating muscle inflammation in combination with [^18^F]-FDG PET/CT imaging findings compatible with systemic vasculitis as well as the clinical diagnosis of systemic vasculitis based on the patient’s chart.

**Fig. 3:**
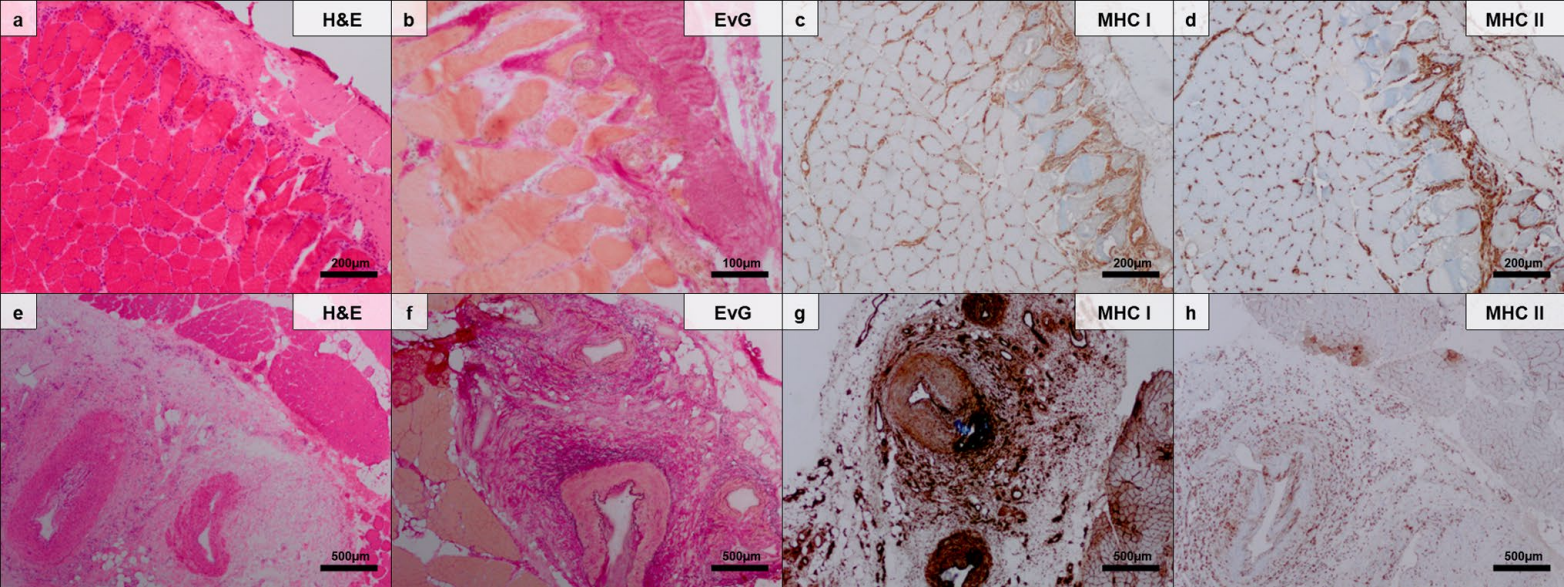
The spectrum of small vessel vasculitis (SVV) at the muscle fascia (M-F) interface (**a**–**d**) and medium vessel vasculitis in the fascia (*fascial vasculitis *sensu stricto) (**e**–**h**) being present in this study is highlighted. SVV features a variably dense lymphomonocytic infiltrate at the muscle–fascia interface (**a**) with edematous changes as well as fibrosis (**b**), while monocytes macrophages are prominently activated in major histocompatibility complex (MHC) class I and MHC class II immunohistochemical stains (**c**, **d**) (hematoxylin and eosin staining, Elastica van Gieson staining, MHC class I and MHC class II immunohistochemistry). Note that the muscle fibers themselves react only minimally, which is at variance with perimysial pathology in dermatomyositis and antisynthetase syndrome. However, prominent inflammation and edematous alterations are present in proper fasciitis affecting larger (medium-sized) arteries and the fascia (**e**, **f**) (hematoxylin and eosin staining, Elastica van Gieson staining). MHC class I staining highlights the prominently activated and widespread macrophages, but also reacts with the perifascicular muscle fibers, showing a gradient toward the centrofascicular regions (**g**) (MHC class I immunohistochemistry). Some muscle fibers close to the vasculitic process also react with MHC class II stains (**h**) (MHC class II immunohistochemistry)

Fasciitis was a prominent feature of our cohort and was observed in all cases (10/10; 100%) in which muscle biopsy specimens contained epimysial fascia. Inflammatory involvement of the fascia mainly affecting small arteries at the muscle–fascia interface or small vessels within the fascia itself (9/10: 90.0%) and was termed ‘vasculitic fasciitis’ (VF) (Fig. 3). As such, VF was defined as vessel wall inflammation located at the (1) muscle–fascia interface or (2) within the fascia. VF at the muscle–fascia interface was characterized by prominent perivascular inflammation of multiple small epimysial arterioles (while features of fibrinoid vessel wall necrosis and fragmentation of nuclei were absent). VF within the fascia itself showed involvement of larger arterioles. Both phenotypes showed perifocal inflammatory infiltrates (around the inflamed vessel itself).

One case showed fasciitis indicated by inflammatory infiltrates within the fascia without signs of vasculitic involvement.

Mild endomysial fibrosis and muscle fiber necrosis were noted in about half (6/12; 50.0% and 7/12; 58.3%) of the muscle biopsy specimens, respectively.

Sarcolemmal staining of myofibers for MHC class I was observed in 10/11 (90.9%) cases, while focal MHC class II upregulation on myofibers was detected in 2/12 (16.6%) cases. Vascular upregulation of PDGFRB on pericapillary pericytes with enlarged capillary lumina was observed in all cases (10/10; 100%), and this feature was pronounced close to the vasculitis areas more prominently than at a distance.

Localization of predominant inflammatory infiltrates was equally distributed among the different muscle compartments. Semiquantitative analysis of the inflammatory infiltrates revealed at least moderate presence of CD68^+^ macrophages in 8/11 (72.7%) cases, respectively. The loss of CD31^+^ endothelial cells highlighted the destruction of capillaries in the perifocal areas.

Figures 1, 3, and Tables 3, 4 display the histopathologic features of the analyzed muscle biopsy specimens.

**Table 3:** Histopathologic features of ANCA-negative vasculitic myopathy in the Berlin cohort

Predominant localization of inflammatory infiltrates	
Epimysial inflammation	4/12 (33.3%)
Perimysial inflammation	4/12 (33.3%)
Endomysial inflammation	4/12 (33.3%)
Skeletal muscle vasculitis	8/12 (66.6%)
Features of necrotizing vasculitis	3/12 (25.0%)
Medium vessel vasculitis	3/12 (25.0%)
Small vessel vasculitis	6/12 (50.0%)
Perivascular infiltrates	10/12 (83.3%)
Fasciitis	10/10 (100%)
Vasculitic fasciitis	9/10 (90.0%)
Vessel occlusion	7/12 (58.3%)
Muscle fiber necrosis	7/12 (58.3%)
Variation of muscle fiber size	12/12 (100%)
Signs of neurogenic damage	7/12 (58.3%)
Capillary pathology	
Complement deposits	2/12 (16.6%)
Capillary vessel mural thickening	12/12 (100%)
Endomysial fibrosis	6/12 (50%)
Major histocompatibility complex class I upregulation on myofibers	10/11 (90.9%)
Major histocompatibility complex class II upregulation on myofibers (focal positivity)	2/12 (16.6%)
Vascular upregulation of platelet-derived growth factor receptor beta	10/10 (100%)

**Table 4:** Semiquantitative and quantitative analysis of histopathologic features of ANCA-negative vasculitic myopathy in the Berlin cohort

Semiquantitative analysis	
CD31^+^ cells	− 0/10; + 4/10; ++ 1/10; +++ 5/10
CD68^+^ cells	− 0/11; + 3/11; ++ 3/11; +++ 5/11
Quantitative analysis of inflammatory infiltrates	
Mean (sd) CD3^+^ lymphocytes count per HPF	11.3 (10.5)
Mean (sd) CD8^+^ lymphocytes count per HPF	6.3 (5.1)
Mean (sd) CD45^+^ lymphocytes count per HPF	18.1 (17.1)

Semiquantitative analysis was performed as follows: + mildly positive/present; ++ moderately positive/present; +++ strongly positive/present; − negative/not present

*sd* standard deviation

### Ultrastructural features of ANCA-negative vasculitic myopathy

**Fig. 4:**
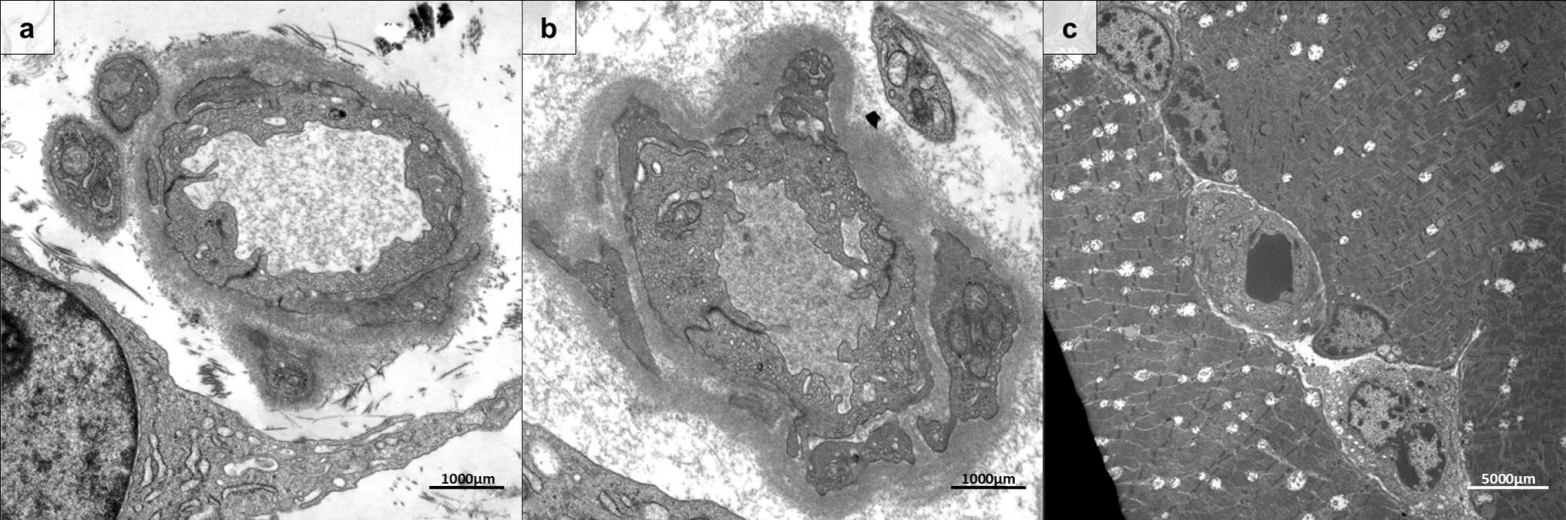
Ultrastructural features of vasculitic myopathy in ANCA-negative vasculitis. Capillary pathology includes prominent basement membrane thickening and some pericyte processes within the basement membrane. The endothelium contains multiple small vesicles, indicating activation of endothelial cells. A fibroblast with an elongated process seems to ‘embrace’ the capillary in addition to signs of activation such as rough endoplasmic reticulum (**a**). Endothelial activation as well as basement membrane thickening involving proliferation of pericytes is observed in a capillary (**b**). At lower magnification, an enlarged capillary with two lymphomonocytic cells in proximity is evident (**c**)

Muscle biopsy specimens from two patients were analyzed at the subcellular level. Ultrastructural features of ANCA-negative VM featured signs of capillary pathology, including basement membrane thickening and endothelial activation indicated by the accumulation of small vesicles and rough endoplasmic reticulum (Fig. 4).

### Gene expression in ANCA-negative vasculitic myopathy by RT-qPCR

**Fig. 5:**
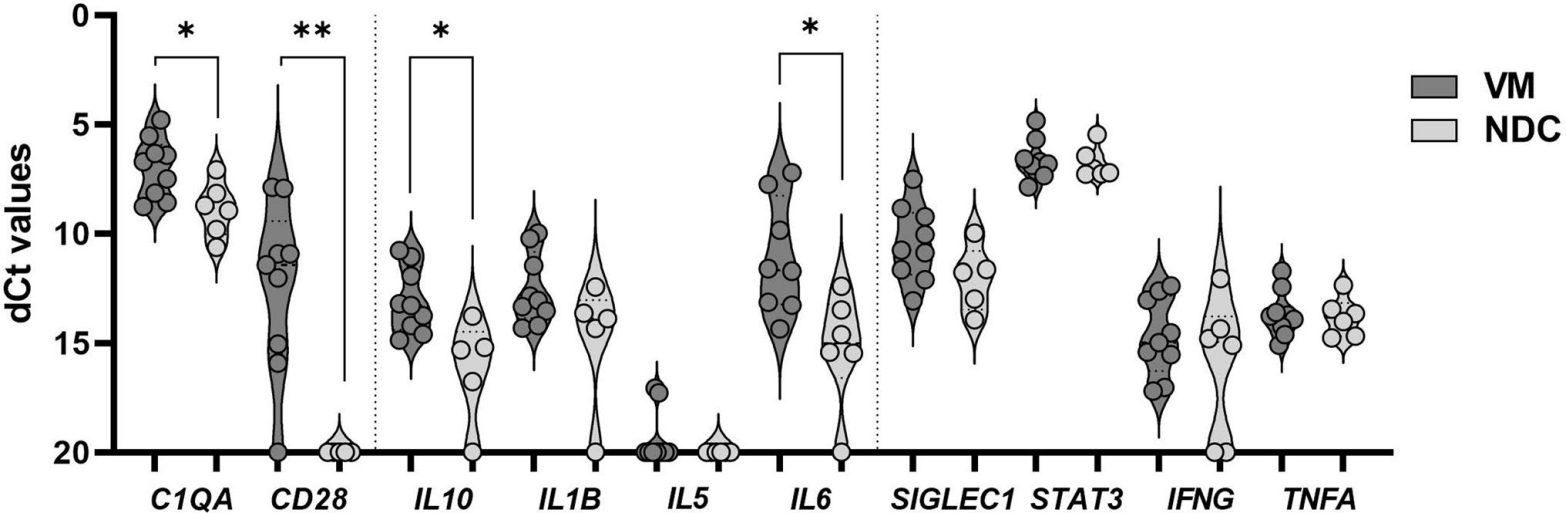
Analysis of gene expression by qRT-PCR in ANCA-negative vasculitic myopathy (Berlin cohort) demonstrated a significant upregulation of *C1QA*, *CD28*, *IL10,* and *IL6* indicating a broad activation of proinflammatory pathways. Visualization of dCt values showing expression of vasculitic myopathy and non-disease controls. Mann–Whitney *U* test, *p* < 0.05. *NDC* non-disease controls, *VM* vasculitic myopathy

Analysis of gene expression showed a significant upregulation of *C1QA*, *CD28*, *IL10*, and *IL6* compared to NDC, while the gene expression of *IFNG*, *IL1B*, *SIGLEC1*, *STAT3*, and *TNFA* did not differ between the groups, and *IL5* was not expressed in NDC or ANCA-negative VM (Fig. 5). These results suggest the involvement of the classical complement pathway, T-cell co-stimulatory pathways, and broad immunomodulatory pathways by activation (via IL-6) and downregulation (via IL-10).

### Gene expression in ANCA-negative vasculitic myopathy by RNA sequencing

This study included one muscle biopsy specimen from each of 36 patients with ANCA-negative VM, drawn from two independent cohorts (Berlin cohort and Barcelona cohort), and compared to 37 control biopsies and 649 samples from other forms of IIM (Supplementary Table S2). The transcriptional profiles of the two ANCA-negative VM cohorts were highly similar (Supplementary Fig. S3).

**Fig. 6:**
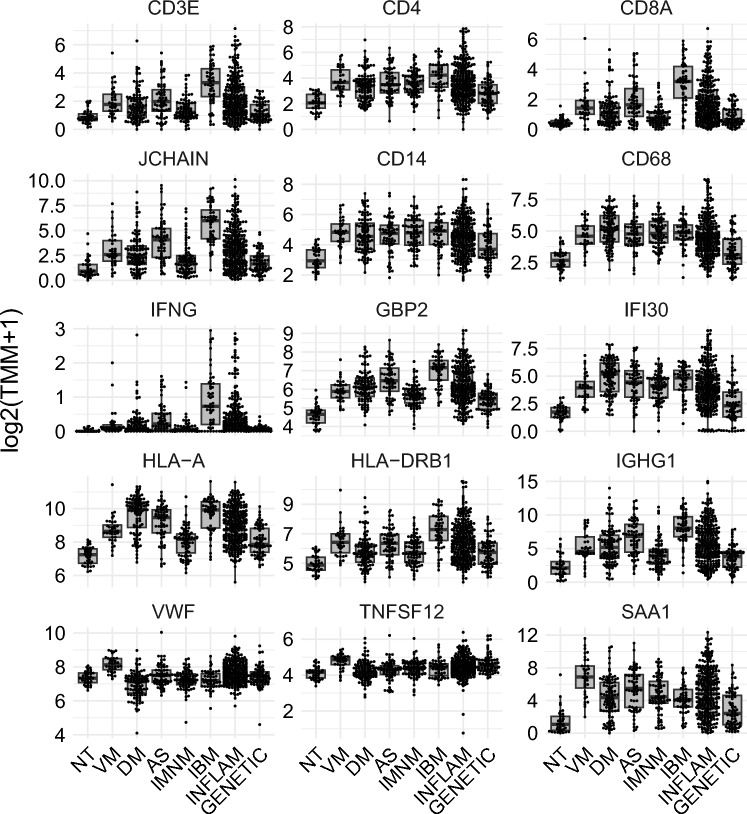
Expression of representative genes in ANCA-negative vasculitic myopathy compared to other myopathies. Each dot represents the gene expression value of a single patient. The expression profile of VM is characterized by upregulation of interferon-gamma-inducible genes, inflammatory markers including *SAA1*, elevated expression of *VWF* and certain TNF ligands such as *TNFSF12*. *NT *histologically normal muscle biopsies, *VM* vasculitic myopathy, *DM* dermatomyositis, *AS* antisynthetase syndrome, *IMNM* immune-mediated necrotizing myopathy, *IBM* inclusion body myositis, *INFLAM* inflammatory myopathies, *GENETIC* genetic myopathies

**Fig. 7:**
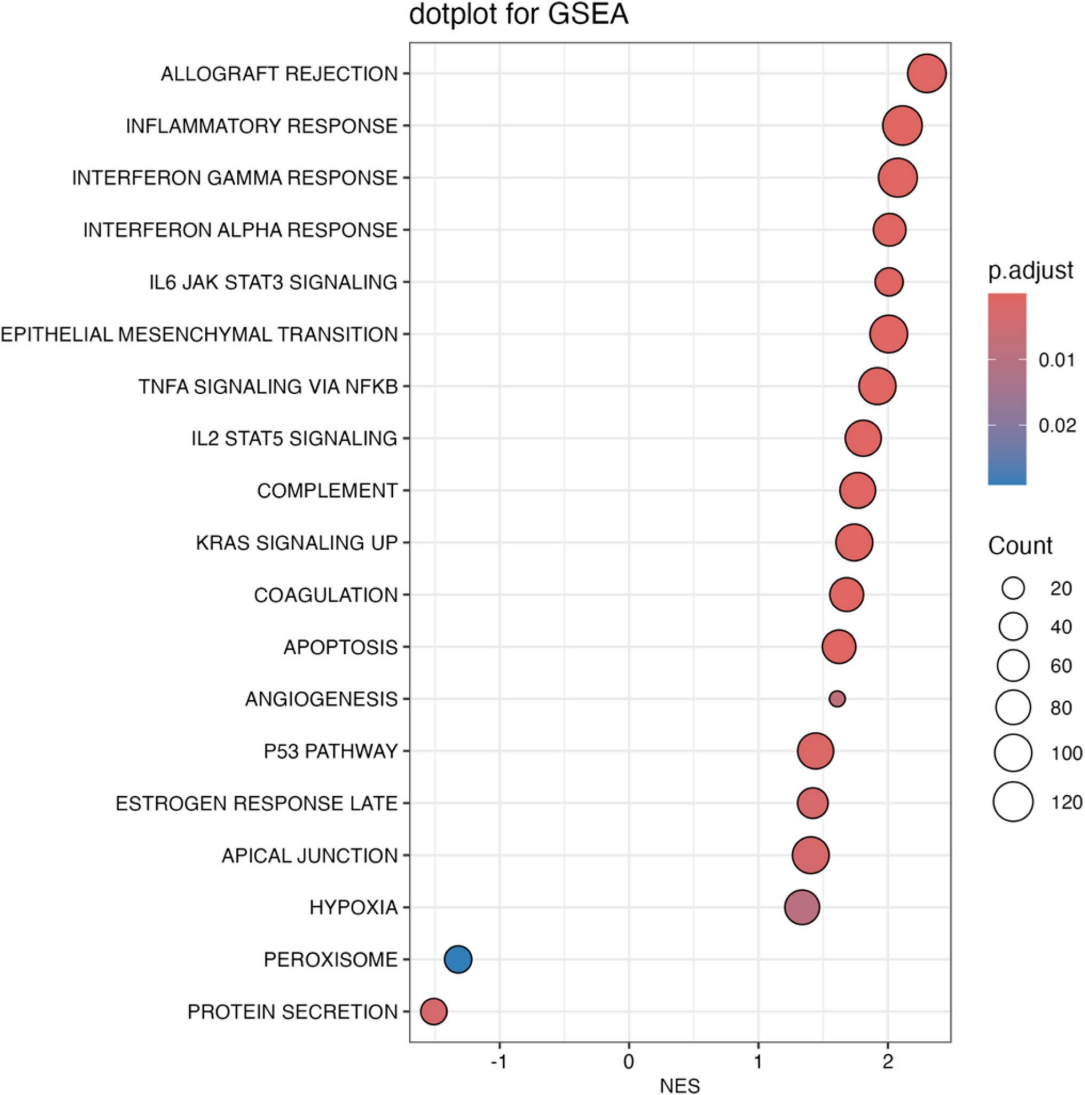
Gene Set Enrichment Analysis of the hallmark gene sets from the Human Molecular Signatures Database in patients with ANCA-negative vasculitic myopathy (VM) compared to normal muscle*.* Pathway analysis and gene expression profiling of VM muscle biopsies exhibited a marked upregulation of interferon-gamma-inducible genes, with expression levels comparable to those observed in dermatomyositis. *NES* normalized enrichment score*;* *p* adjust*,* adjusted *p* value

Pathway analysis and gene expression profiling of ANCA-negative VM muscle biopsies exhibited a marked upregulation of interferon-gamma (IFNγ)-inducible genes, with expression levels similar in magnitude (but different in nature) to those observed in dermatomyositis (DM) (Figs. 6, 7, Supplementary Fig. S4, Supplementary Fig. S5). In contrast, type I interferon-inducible genes, such as *ISG15* and *MX1*, were only mildly elevated, slightly higher than in immune-mediated necrotizing myositis (IMNM) (Supplementary Fig. S4, Supplementary Fig. S5). The transcriptomic inflammatory marker *SAA1* was particularly elevated in ANCA-negative VM compared to other muscle biopsy samples (Fig. 6).

T-cell antigens showed moderate upregulation, with a relative predominance of *CD4* over *CD8* expression (Fig. 6, Supplementary Fig. S6). Plasma cell markers (*JCHAIN*, *SDC1*) were elevated to levels comparable to DM, and there was increased expression of various immunoglobulin isotypes (Supplementary Fig. S6, Supplementary Fig. S7). Macrophage markers (*CD14*, *CD68*) were also upregulated, at levels similar to those seen in other inflammatory myopathies (Supplementary Fig. S6). In addition, angiogenesis-related pathways were enriched, and the canonical endothelial cell marker *VWF* was significantly elevated in ANCA-negative VM compared to other myopathies. In contrast, the venular endothelial marker *ACKR1* was expressed at levels similar to those seen in IIM subtypes (Fig. 6, Supplementary Fig. S6).

HLA class I genes were mildly upregulated in ANCA-negative VM, slightly exceeding levels seen in IMNM. In contrast, HLA class II genes typically induced by T cell-derived IFNγ were more strongly upregulated in ANCA-negative VM than in DM, comparable to those in antisynthetase syndrome (AS) and lower than in inclusion body myositis (IBM) (Supplementary Fig. S7).

Expression of type IIA myofiber marker *MYH2* was more downregulated than markers of other fiber types (MYH7, MYH1) (Supplementary Fig. S8). Structural muscle genes (*ACTA1*, *TTN*) were reduced to levels similar to those in DM, while markers of muscle regeneration (*NCAM1*, *PAX7*, *MYH3*, *MYH8*) were only mildly increased compared to normal muscle, and lower than in other inflammatory myopathies. Mitochondrial genes showed a global reduction, a pattern consistent with other inflammatory myopathies (Supplementary Fig. S9).

Interleukin gene expression analysis revealed strong upregulation of multiple interleukins, including *IL34*, *IL17B*, *IL16*, *IL7*, *IL6*, and *IL8* (Supplementary Fig. S10). A similar trend was observed for interleukin receptors such as *IL4R*, *IL10RA*, *IL17RA*, *IL21R*, and *IL2RA* (Supplementary Fig. S11). Checkpoint ligands and their corresponding receptors were also upregulated (Supplementary Fig. S12).

Chemokine-encoding genes—including *CCL4*, *CCL3*, *CCL18*, and *CXCL11*—were upregulated, as were chemokine receptor genes such as the above-mentioned *ACKR1*, *CCR1*, *CXCR6*, and *CCR5* (Supplementary Fig. S13, Supplementary Fig. S14). Several tumor necrosis factor (TNF) superfamily genes (*TNFSF8*, *LTB*, *TNFSF12*, *TNFSF13B*, *TNFSF14*, *TNFSF13*, *EDA*) were upregulated, with *LTB* and *EDA* reaching higher levels than in other inflammatory myopathies (Supplementary Fig. S15). TNF receptor genes—including *TNFRSF18*, *TNFRSF14*, and *TNFRSF4*—were also elevated (Supplementary Fig. S16).

*TGFB1* and *TGFB3* were expressed at levels similar to other inflammatory myopathies, while their receptors—particularly those for *TGFB2* and *TGFB3*—were more strongly upregulated in ANCA-negative VM than in other IIM subtypes (Supplementary Fig. S17).

## Discussion

VM is a neglected organ manifestation that can develop in various vasculitis syndromes. The stereotypical presentation of VM comprises myalgia, muscle weakness, constitutional symptoms, and elevated inflammatory markers indicating systemic inflammation. However, this clinical pattern is nonspecific and can be present in various clinical contexts including IIM.

Notably, MRI studies are currently unable to reliably distinguish VM from primary muscle inflammation (“myositis”), and muscle biopsy still represents the gold standard for the reliable diagnosis of both entities. The differentiation between VM and myositis is of great interest to clinicians, as these entities clearly differ in pathophysiology, organ manifestation, clinical course, and, therefore, management [55].

State-of-the-art management of immune-mediated diseases includes signature cytokine-based treatment approaches [59], which are crucial in rare diseases where data from randomized controlled trials are lacking, and clinicians must develop treatment strategies based on the current understanding of disease pathophysiology. From this perspective, our study provides a framework that confirms the findings of previous studies on VM and offers new insights into the histomorphology and immunopathogenesis of VM in ANCA-negative vasculitis, which may guide future diagnosis and treatment.

Our results validate the previously reported clinical presentation of VM that is characterized by myalgia, predominant distal lower extremity involvement, normal serum CK activity, highly elevated inflammatory markers, and T2-hyperintensities on MRI [27]. The systemic nature of VM in our cohort is also reflected by commonly reported extramuscular symptoms such as arthralgia or skin lesions and the morphologic evidence of concomitant neuropathy. In line with previous findings from [^18^F]-FDG PET imaging studies in PAN [13, 14, 42, 55], we observed a similar pattern of pathologic radiotracer uptake within the musculature and peri-/intramuscular arteries that suggests the presence of VM.

Systematic phenotyping was performed on the histomorphologic level. ANCA-negative VM frequently affected small arteries and presented less frequently as MVV [27]. Consistent with the recent results from a large cohort study [27], necrotizing vasculitis was noted in a subset of muscle biopsy specimens. The frequent observation of vasculitic inflammation at the muscle–fascia interface, in addition to vasculitis within the fascia itself, is termed ‘vasculitic fasciitis’, representing a hallmark of our study that has been occasionally reported in single cases [19, 22, 23, 36, 40]. However, the potential diagnostic value of this distinct histopathological pattern had not been recognized so far. Our study suggests that VF constitutes a characteristic lesion of ANCA-negative VM that may have been undiagnosed in the past due to the lack of fascial tissue in muscle biopsies. MRI signs of fascial inflammation in VM have also been described by others [1, 17, 23, 36, 40, 61, 62], but a corresponding histopathologic investigation was lacking in the vast majority of cases. To the best of our knowledge, VF has not been reported in clinical contexts other than VM. Notably, the observed pattern of VF seen in our cohort is distinct from fasciitis seen in other inflammatory conditions such as AS or eosinophilic fasciitis (Supplementary Fig. S19, Supplementary Fig. S20, Supplementary Table S21) [41]. The characteristic morphology of VF supports the routine acquisition of fascial tissue during muscle biopsy procedures for suspected inflammatory myopathy, because the confirmation of VF could aid the diagnosis of ANCA-negative VM in cases where skeletal muscle vasculitis may be missed due to ‘skip lesions’ or sampling error (but imaging studies still indicate muscle inflammation). The presence of VF and concomitant absence of sarcolemmal positivity for MHC class II may also help to distinguish ANCA-negative VM from specific IIM subtypes (e.g., overlap myositis, IBM, immune checkpoint inhibitor-related myositis) that do not exhibit fasciitis on the histomorphologic level, but feature sarcolemmal upregulation of MHC class II [37]. Finally, VF presumably contributes to localized pain reported by the patients and may be included in future concepts of pathophysiology [36].

In contrast to certain IIM subtypes, the distribution of inflammatory infiltrates in ANCA-negative VM showed no specific pattern within the muscle compartments. The generally mild degree of endomysial fibrosis and muscle fiber necrosis in ANCA-negative VM could be compatible with a rather acute disease onset and aligns with normal or mildly elevated levels of serum CK activity noted in this and previous studies, respectively [27]. The pronounced MHC class I upregulation on the histomorphologic level seen in our study could reflect our cohort’s homogeneity, contrasting with a larger study [27] that included a substantial proportion of ANCA-positive cases. This difference supports the notion of distinctive VM phenotypes based on ANCA status.

Analysis of gene expression demonstrated a strong upregulation of *IL6* that provides a pathophysiological rationale for the application of tocilizumab (TCZ) and sarilumab, two interleukin (IL)-6 antagonists, in ANCA-negative VM. This notion is of special interest, since half of the patients from the Berlin cohort met the 1990 ACR classification criteria for ‘classic PAN’. Notably, previous studies have reported relatively high rates of remission in patients with refractory PAN who were treated with TCZ [18, 29, 31, 67]. Corresponding to our results and the mentioned studies, a correlation of serum IL-6 levels and disease activity has been described in patients with cutaneous PAN [29].

The transcriptomic analysis revealed that ANCA-negative VM is defined by a dominant IFNγ-driven signature—evident from pathway analysis and the elevation of IFNγ-inducible genes such as *GBP2* and *IFI30*—accompanied by selective upregulation of endothelium- and angiogenesis-related genes, including *vWF*, likely reflecting ongoing vascular injury consistent with its vasculitic nature. Capillary vessel mural thickening on the histomorphologic level and signs of endothelial activation demonstrated by ultrastructural analysis align with these findings. Reports of increased serum levels of von Willebrand factor (vWF) in active vasculitis also strengthen this notion [25]. Immune cell infiltration included CD4-skewed T-cell signatures [8], elevated plasma cell and macrophage markers, and marked upregulation of HLA class II genes. Despite the pronounced immune activation, muscle fiber damage was relatively limited, characterized by modest downregulation of structural and type IIA fiber genes, reduced regenerative markers, and global mitochondrial suppression. Broad activation of cytokine networks—including *IL-6* and other interleukins, chemokines, and TNF superfamily members—further highlights the complex inflammatory milieu in ANCA-negative VM. The reported T-cell signature and upregulation of TNF receptor genes raise the question of whether targeting Janus kinase–signal transducer and activator of transcription (JAK/STAT) signaling may be effective in PAN-associated VM [52, 53]. Finally, the observed upregulation of *SAA1* corresponds to biomarker studies of serum amyloid A in various vasculitides [64]. However, ANCA-negative VM has not been studied in this context.

The strengths of our study comprise the multidimensional phenotyping, including histomorphology, ultrastructural analysis, and gene expression analysis of a neglected condition in a homogeneous cohort of ANCA-negative patients. Specifically, the transcriptomic profiles in our two cohorts were highly similar, suggesting a shared endotype despite inconsistent nomenclature and definitions of VM reported in the literature, thereby enhancing the generalizability of our findings. Results from the gene expression analysis provide the underpinning for future studies of pathophysiology and therapeutic approaches.

The present study has several limitations, including the small sample size and retrospective design, which pose a risk of bias and may influence the external validity of the results. For example, retrospective analyses rely on previously recorded data, which can be incomplete or inconsistently documented, possibly limiting the accuracy and depth of clinical correlations. The lack of detailed data on immunosuppression at the time of muscle biopsy reflects this issue. Furthermore, genetic testing for deficiency of adenosine deaminase 2 (DADA2), a young-onset PAN mimic that is characterized by a type 1 interferon signature and TNF dysregulation [6, 10], was not available. Notably, rare cases of DADA2-associated VM have also been reported [4, 7, 12, 33, 35, 69].

In summary, VM in ANCA-negative vasculitis is a condition characterized by myalgia, skeletal muscle vasculitis of small arteries that predominantly involves the lower extremities. Inflammatory markers are highly elevated, while serum CK activity is normal or mildly elevated. MRI can demonstrate T2-hyperintensities indicating muscle edema secondary to skeletal muscle vasculitis (‘MRI myositis sine myositis’) that warrants biopsy confirmation. VF represents a distinct histopathological pattern and possibly a diagnostic lesion associated with ANCA-negative VM that can manifest with signs of fascial inflammation on MRI and likely contributes to localized pain. ANCA-negative VM is characterized by a dominant IFNγ-driven immune response, broad cytokine activation including IL-6, TNF-related genes, and selective upregulation of angiogenesis- and endothelium-associated transcripts.

## Supplementary Information

Below is the link to the electronic supplementary material.

Supplementary file1 (DOCX 10825 KB)

## Data Availability

All data relevant to the study are included in the article or uploaded as online supplemental material. No further data are available.
