# Supplementary material for: Vasculitic fasciitis characterizes a distinct subset of vasculitic myopathy with interferon-gamma signature
Source: Acta Neuropathol. 2025 Dec 24;151(1):2. doi: 10.1007/s00401-025-02969-1 (PMC12738632; doi:10.1007/s00401-025-02969-1)
Supplement: Supplementary file 1 — Supplementary file1 (DOCX 10825 KB) [file 401_2025_2969_MOESM1_ESM.docx]

**Vasculitic Fasciitis Characterizes a Distinct Subset of Vasculitic Myopathy with Interferon-gamma Signature**

Nikolas Ruffer^1,2,‡^, Iago Pinal-Fernandez^3,4,‡^, Corinna Preusse^2,5,6^, Andrew L. Mammen^3,4,7^,
Marie-Therese Holzer^1,2^, Felix Kleefeld^5,8,9^, Hans-Hilmar Goebel^2^, Maria Casal-Dominguez^3,4^, Katherine Pak^3^, Ina Kötter^1^, , Jeffrey Siefert^10^, Christian Furth^10^, Felix Feldhaus^11^, Norman Görl^12,13^, Franziska Fieber^12^, Rieke Alten^14^, Tobias B. Huber^1^, Vincent Casteleyn^15^, Andreas Roos^16^, Martin Krusche^1^, Udo Schneider^15,17^, José César Milisenda^18,#^ and Werner Stenzel^2,#,^*

^1^University Medical Center Hamburg-Eppendorf, III. Department of Medicine, Hamburg, Germany.

^2^Charité – Universitätsmedizin Berlin, corporate member of Freie Universität Berlin and Humboldt-Universität zu Berlin, Department of Neuropathology, Berlin, Germany.

^3^National Institutes of Health, National Institute of Arthritis and Musculoskeletal and Skin Diseases, Bethesda, Muscle Disease Section, Bethesda, Maryland, United States.

^4^Johns Hopkins University, School of Medicine, Department of Neurology, Baltimore, Maryland, United States.

^5^Charité – Universitätsmedizin Berlin, corporate member of Freie Universität Berlin and Humboldt-Universität zu Berlin, Clinic for Neurology with Experimental Neurology, Berlin, Germany.

^6^Charité – Universitätsmedizin Berlin, corporate member of Freie Universität Berlin and Humboldt-Universität zu Berlin, Clinic for Pediatrics with Neurology, Berlin, Germany.

^7^Johns Hopkins University, School of Medicine, Department of Medicine, Baltimore, Maryland, USA.

^8^Ruhr University Bochum, University Hospital Bergmannsheil, Department of Neurology, Heimer Institute for Muscle Research, Bochum, Germany.

^9^Ruhr University Bochum, BG University Hospital Bergmannsheil, Department of Neurology, Bochum, Germany

^10^Charité – Universitätsmedizin Berlin, Corporate Member of Freie Universität Berlin and Humboldt-Universität zu Berlin, Department of Nuclear Medicine, Berlin Germany.

^11^Charité – Universitätsmedizin Berlin, corporate member of Freie Universität Berlin and Humboldt-Universität zu Berlin, Department of Radiology, Berlin, Germany.

^12^Rheumazentrum Nordwest, Wismar, Germany.

^13^Klinikum Südstadt Rostock, Department of Internal Medicine, Rheumatology and Immunology, Rostock, Germany.

^14^Department of Internal Medicine and Rheumatology, Schlosspark-Klinik, Berlin, Germany.

^15^Charité – Universitätsmedizin Berlin, corporate member of Freie Universität Berlin and Humboldt-Universität zu Berlin, Department of Rheumatology and Clinical Immunology, Berlin, Germany.

^16^University of Duisburg-Essen, University Children's Hospital Essen, Department of Neuropediatrics, Developmental Neurology and Social Pediatrics, Centre for Neuromuscular Disorders in Children, Essen, Germany.

^17^Immanuel Hospital Berlin, Department of Rheumatology, Clinical Immunology and Osteology, Berlin, Germany.

^18^Hospital Clínic de Barcelona, Internal Medicine Department, Muscular and Inherited Metabolic Disorders Research Laboratory, Barcelona, Spain.

***Correspondence to:** Werner Stenzel, Department of Neuropathology, Charité – Universitätsmedizin Berlin, Charitéweg 1 – Virchowweg 15, 10117 Berlin, Germany. E-mail: [werner.stenzel@charite.de](mailto:werner.stenzel@charite.de)

^‡^Nikolas Ruffer and Iago Pinal-Fernandez share first authorship.

^#^José César Milisenda and Werner Stenzel share last authorship.

## SUPPLEMENTAL MATERIAL

Supplementary Table S1 Summary of primary antibodies used in the study with name, host, clone/code no., dilution, and provider.

| Primary antibody | Company | Dilution | Species |
| --- | --- | --- | --- |
| CD4 | Zymed, BRB042 | 1:100 | rabbit |
| CD8 | DAKO, M7050 | 1:100 | mouse |
| CD20 | DAKO, M0755 | 1:200 | mouse |
| CD31 | DAKO, M0823 | 1:100 | mouse |
| CD45 | DAKO, M0701 | 1:400 | mouse |
| CD68 | DAKO, M0718 | 1:100 | mouse |
| CD138 | DAKO, M7228 | 1:30 | mouse |
| C5b-9 | DAKO, M0777 | 1:200 | mouse |
| MHC class I | DAKO, M0736 | 1:1.000 | mouse |
| MHC class II | DAKO, M0775 | 1:100 | mouse |
| PDGFRB | Santa Cruz, SC-339 | 1:30 | rabbit |
| VEGF | Santa Cruz, SC-7269 | 1:20 | mouse |
| MUM1 | DAKO, M7259 | 1:50 | mouse |
| ICAM, CD54 | DAKO, M7063 | 1:50 | mouse |

**Supplementary Table S2** Groups of patients included for RNA sequencing in the study.

| Group | N = 722^1^ |
| --- | --- |
| Patient group |  |
| Normal biopsies | 37 (5.1%) |
| ANCA-negative vasculitic myopathy | 36 (5.0%) |
| Dermatomyositis  Anti-Mi2  Anti-NXP2  Anti-TIF1g  Anti-MDA5  No known autoantibody | 105 (15%)  22 (3%)  21 (2.9%)  28 (3.9%)  11 (1.5%)  23 (3.2%) |
| Antisynthetase syndrome  Anti-Jo1  Other antisynthetase syndrome autoantibodies | 66 (9.1%)  38 (5.3%)  28 (3.9%) |
| Immune-mediated necrotizing myopathy  Anti-HMGCR  Anti-SRP | 80 (11%)  60 (8.3%)  20 (2.8%) |
| Inclusion body myositis | 53 (7.3%) |
| Other inflammatory myopathies | 273 (38%) |
| Genetic myopathies | 72 (10.0%) |
| Patient cohort for vasculitic myopathy  Berlin  Barcelona | 10 (1.4%)  26 (3.6%) |

**Supplementary Figure S3** Expression of representative genes in ANCA-negative vasculitic myopathy compared to other myopathies, stratified by cohort location. Each dot represents the gene expression value of a single patient. NT*,* histologically normal muscle biopsies; VM*,* vasculitic myopathy*;* DM*,* dermatomyositis; AS*,* antisynthetase syndrome*;* IMNM*,* immune-mediated necrotizing myopathy; IBM*,* inclusion body myositis; INFLAM*,* inflammatory myopathies; GENETIC*,* genetic myopathies.

**Supplementary Fig. S4** Expression of interferon genes in ANCA-negative vasculitic myopathy compared to other myopathies. Each dot represents the gene expression value from a single patient. NT*,* histologically normal muscle biopsies; VM*,* vasculitic myopathy*;* DM*,* dermatomyositis; AS*,* antisynthetase syndrome*;* IMNM*,* immune-mediated necrotizing myopathy; IBM*,* inclusion body myositis; INFLAM*,* inflammatory myopathies; GENETIC*,* genetic myopathies.

**Supplementary Fig. S5** Expression of type 1 and type 2 interferon-inducible genes and interferon receptors in ANCA-negative vasculitic myopathy compared to other myopathies. Each dot represents the gene expression value from a single patient. NT*,* histologically normal muscle biopsies; VM, vasculitic myopathy*;* DM*,* dermatomyositis; AS*,* antisynthetase syndrome*;* IMNM*,* immune-mediated necrotizing myopathy; IBM*,* inclusion body myositis; INFLAM*,* inflammatory myopathies; GENETIC*,* genetic myopathies.

**Supplementary Fig. S6** Expression of transcriptomic markers associated with various cell types in ANCA-negative vasculitic myopathy compared to other myopathies. Markers include T-cell markers (*CD3E*, *CD4*, *CD8A*), B-cell markers (*CD19*, *MS4A1*), plasma cell markers (*SDC1*, *JCHAIN*), neutrophil markers (*ELANE*), macrophage markers (*CD14*, *CD68*), and endothelial cell markers (*VWF*, *ACKR1*). Each dot represents the gene expression value from a single patient. NT*,* histologically normal muscle biopsies; VM, vasculitic myopathy*;* DM*,* dermatomyositis; AS*,* antisynthetase syndrome*;* IMNM*,* immune-mediated necrotizing myopathy; IBM*,* inclusion body myositis; INFLAM*,* inflammatory myopathies; GENETIC*,* genetic myopathies.

**Supplementary Fig. S7** Expression of transcriptomic markers for HLA and various immunoglobulin isotypes in ANCA-negative vasculitic myopathy compared to other myopathies. Each dot represents the gene expression value from a single patient. NT*,* histologically normal muscle biopsies; VM*,* vasculitic myopathy*;* DM*,* dermatomyositis; AS*,* antisynthetase syndrome*;* IMNM*,* immune-mediated necrotizing myopathy; IBM*,* inclusion body myositis; INFLAM*,* inflammatory myopathies; GENETIC*,* genetic myopathies.

**Supplementary Fig. S8** Expression of transcriptomic markers for mature muscle fibers (*MYH7*, *MYH2*, *MYH1*, *ACTA1*, *TTN*) and regenerating muscle fibers (NCAM1, PAX7, MYH3, MYH7) in ANCA-negative vasculitic myopathy compared to other myopathies. Each dot represents the gene expression value from a single patient. NT*,* histologically normal muscle biopsies; VM*,* vasculitic myopathy*;* DM*,* dermatomyositis; AS*,* antisynthetase syndrome*;* IMNM*,* immune-mediated necrotizing myopathy; IBM*,* inclusion body myositis; INFLAM*,* inflammatory myopathies; GENETIC*,* genetic myopathies.

**Supplementary Fig. S9** Expression of mitochondrial transcriptomic markers in ANCA-negative vasculitic myopathy compared to other myopathies. Each dot represents the gene expression value from a single patient. NT*,* histologically normal muscle biopsies; VM*,* vasculitic myopathy*;* DM*,* dermatomyositis; AS*,* antisynthetase syndrome*;* IMNM*,* immune-mediated necrotizing myopathy; IBM*,* inclusion body myositis; INFLAM*,* inflammatory myopathies; GENETIC*,* genetic myopathies.

**Supplementary Fig. S10** Expression of significantly different (q-value < 0.05 compared to histologically normal muscle biopsies) interleukin genes in ANCA-negative vasculitic myopathy compared to other myopathies. Each dot represents the gene expression value from a single patient. NT*,* histologically normal muscle biopsies; VM*,* vasculitic myopathy*;* DM*,* dermatomyositis; AS*,* antisynthetase syndrome*;* IMNM*,* immune-mediated necrotizing myopathy; IBM*,* inclusion body myositis; INFLAM*,* inflammatory myopathies; GENETIC*,* genetic myopathies.

**Supplementary Fig. S11** Expression of significantly different (q-value < 0.05 compared to histologically normal muscle biopsies) interleukin receptor genes in ANCA-negative vasculitic myopathy compared to other myopathies. Each dot represents the gene expression value from a single patient. NT*,* histologically normal muscle biopsies; VM*,* vasculitic myopathy*;* DM*,* dermatomyositis; AS*,* antisynthetase syndrome*;* IMNM*,* immune-mediated necrotizing myopathy; IBM*,* inclusion body myositis; INFLAM*,* inflammatory myopathies; GENETIC*,* genetic myopathies.

**Supplementary Fig. S12** Expression of checkpoint inhibitor ligands and receptors in ANCA-negative vasculitic myopathy compared to other myopathies. Each dot represents the gene expression value from a single patient. NT*,* histologically normal muscle biopsies; VM*,* vasculitic myopathy*;* DM*,* dermatomyositis; AS*,* antisynthetase syndrome*;* IMNM*,* immune-mediated necrotizing myopathy; IBM*,* inclusion body myositis; INFLAM*,* inflammatory myopathies; GENETIC*,* genetic myopathies.

**Supplementary Fig. S13** Expression of significantly different (q-value < 0.05 compared to histologically normal muscle biopsies) chemokine genes in ANCA-negative vasculitic myopathy compared to other myopathies. Each dot represents the gene expression value from a single patient. NT*,* histologically normal muscle biopsies; VM*,* vasculitic myopathy*;* DM*,* dermatomyositis; AS*,* antisynthetase syndrome*;* IMNM*,* immune-mediated necrotizing myopathy; IBM*,* inclusion body myositis; INFLAM*,* inflammatory myopathies; GENETIC*,* genetic myopathies.

**Supplementary Fig. S14** Expression of significantly different (q-value < 0.05 compared to histologically normal muscle biopsies) chemokine receptor genes in ANCA-negative vasculitic myopathy compared to other myopathies. Each dot represents the gene expression value from a single patient. NT*,* histologically normal muscle biopsies; VM*,* vasculitic myopathy*;* DM*,* dermatomyositis; AS*,* antisynthetase syndrome*;* IMNM*,* immune-mediated necrotizing myopathy; IBM*,* inclusion body myositis; INFLAM*,* inflammatory myopathies; GENETIC*,* genetic myopathies.

**Supplementary Fig. S15** Expression of significantly different (q-value < 0.05 compared to histologically normal muscle biopsies) tumor necrosis factor genes in ANCA-negative vasculitic myopathy compared to other myopathies. Each dot represents the gene expression value from a single patient. NT*,* histologically normal muscle biopsies; VM*,* vasculitic myopathy*;* DM*,* dermatomyositis; AS*,* antisynthetase syndrome*;* IMNM*,* immune-mediated necrotizing myopathy; IBM*,* inclusion body myositis; INFLAM*,* inflammatory myopathies; GENETIC*,* genetic myopathies.

**Supplementary Fig. S16** Expression of significantly different (q-value < 0.05 compared to histologically normal muscle biopsies) tumor necrosis factor receptor genes in ANCA-negative vasculitic myopathy compared to other myopathies. Each dot represents the gene expression value from a single patient. NT*,* histologically normal muscle biopsies; VM*,* vasculitic myopathy*;* DM*,* dermatomyositis; AS*,* antisynthetase syndrome*;* IMNM*,* immune-mediated necrotizing myopathy; IBM*,* inclusion body myositis; INFLAM*,* inflammatory myopathies; GENETIC*,* genetic myopathies.

**Supplementary Fig. S17** Expression of *TGF*$\beta$ genes and receptors in ANCA-negative vasculitic myopathy. Each dot represents the gene expression value from a single patient. NT*,* histologically normal muscle biopsies; VM*,* vasculitic myopathy*;* DM*,* dermatomyositis; AS*,* antisynthetase syndrome*;* IMNM*,* immune-mediated necrotizing myopathy; IBM*,* inclusion body myositis; INFLAM*,* inflammatory myopathies; GENETIC*,* genetic myopathies.

**Supplementary Table S18** Summary of electrodiagnostic findings in the ‘Berlin cohort’.

| **Electromyography**  Myopathic pattern | 4/12 (33.3%)  1/4 (25.0) |
| --- | --- |
| **Nerve conduction study**  Sensorimotor neuropathy  Axonal neuropathy | 6/12 (50.0%)  1/6 (16.6%)  1/6 (16.6%) |


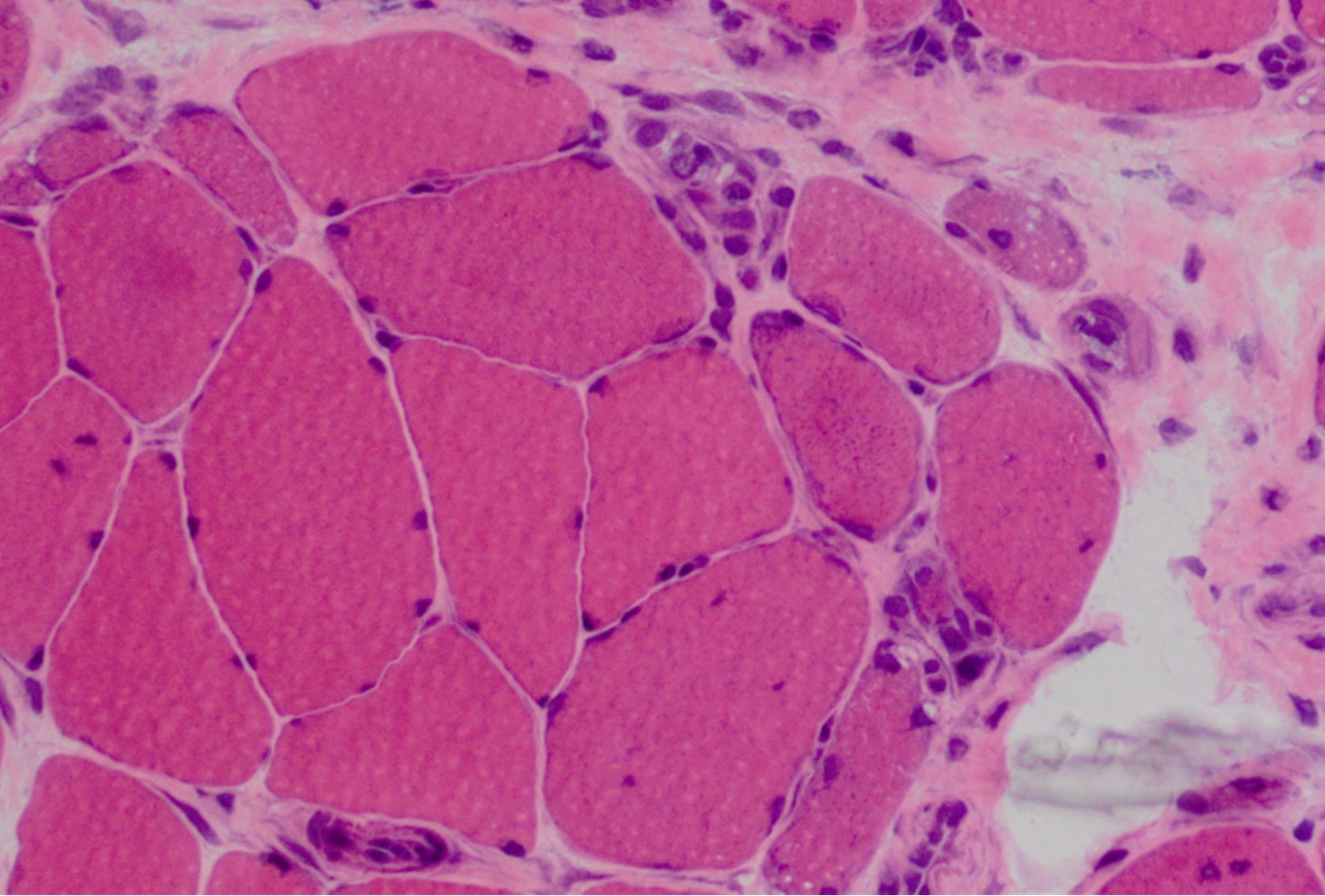


**Supplementary Figure S19** Histomorphology of the muscle-fascia interface in antisynthetase syndrome (hematoxylin and eosin staining). Epimysial small vessels showed no signs of vasculitis such as vessel wall infiltration, vessel wall disruption, fibrinoid necrosis, vessel thrombosis or vessel thrombosis, vessel scarring/fibrosis or vessel wall separation, fragmentation and damage by inflammatory cells.


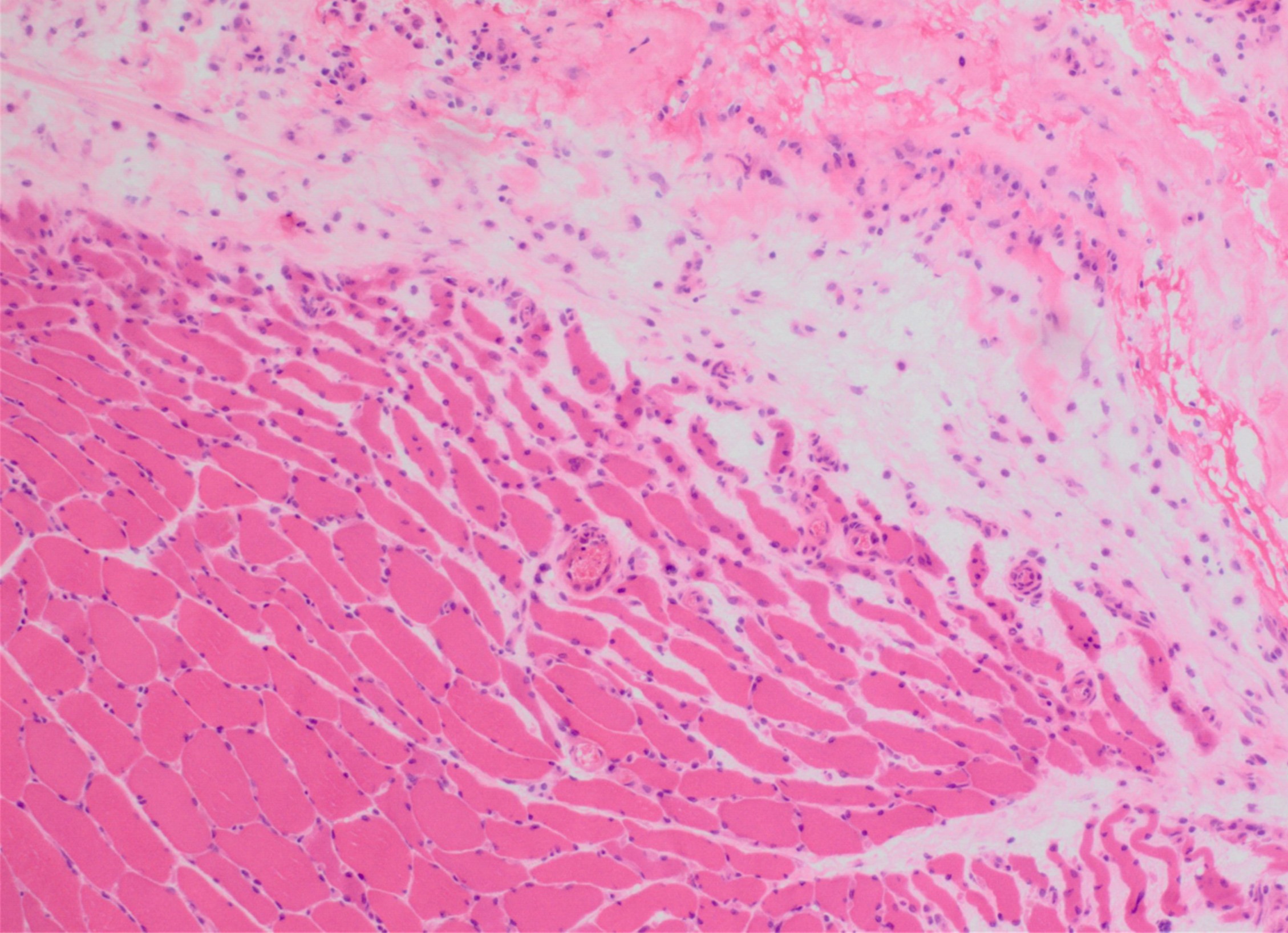


**Supplementary Figure S20** Histomorphology of the muscle-fascia interface in eosinophilic fasciitis (hematoxylin and eosin staining). Epimysial small vessels showed no signs of vasculitis such as vessel wall infiltration, vessel wall disruption, fibrinoid necrosis, vessel thrombosis or vessel thrombosis, vessel scarring/fibrosis or vessel wall separation, fragmentation and damage by inflammatory cells.

**Supplementary Table S21** Comparative evaluation of fascial pathology in antisynthetase syndrome (AS), eosinophilic fasciitis (EF), immune-mediated necrotizing myopathy (IMNM) and healthy controls (HCtrl).

|  | AS  _1 | AS  _2 | AS  _3 | AS  _4 | AS  _5 | EF  _1 | EF  _2 | EF  _3 | EF  _4 | EF  _5 | IMNM  _1 | IMNM  _2 | IMNM  _3 | IMNM  _4 | IMNM  _5 | HCtrl  _1 | HCtrl  _2 | HCtrl  _3 | HCtrl  _4 | HCtrl  _5 | HCtrl  _6 |
| --- | --- | --- | --- | --- | --- | --- | --- | --- | --- | --- | --- | --- | --- | --- | --- | --- | --- | --- | --- | --- | --- |
| Age | 69 | 50 | 67 | 64 | 57 | 50 | 61 | 54 | 67 | 58 | 80 | 77 | 75 | 73 | 59 | 65 | 69 | 38 | 46 | 43 | 42 |
| Sex | F | M | M | M | F | M | M | F | M | F | F | F | F | M | M | M | F | F | F | M | F |
| site | Quad | Delt | Quad | Quad | Quad | Quad | Quad | Quad | Gastroc | Quad | Quad | Quad | Quad | Quad | Quad | Quad | Quad | Quad | Quad | Quad | Quad |
| VF | No | No | No | No | No | No | No | No | No | No | No | No | No | No | No | No | No | No | No | No | No |
| (diffuse) fasciitis | Yes | No | No | Yes | No | Yes | Yes | Yes | Yes | Yes | No | No | No | No | No | No | No | No | No | No | No |

*Note*: Delt, Deltoideus muscle; F, female; Gastroc, Gastrocnemius muscle; M, male; Quad, Quadriceps muscle; VF, vasculitic fasciitis.
